# Venovenous Extracorporeal Membrane Oxygenation (ECMO) as a Life-Saving Intervention in Refractory Status Asthmaticus: A Case Report

**DOI:** 10.7759/cureus.92216

**Published:** 2025-09-13

**Authors:** Apurva Popat, Asem Abu-Jamea, Jayanth Vedre, Delyse Garg, Martin Reriani

**Affiliations:** 1 Internal Medicine, Marshfield Clinic Health System, Marshfield, USA; 2 Internal Medicine, Marshfield Clinic Health System, Marshfield, USA; 3 Critical Care, Marshfield Clinic Health System, Marshfield, USA

**Keywords:** acute severe asthma, extra-corporeal membrane oxygenation, refractory asthma, status asthmaticus, veno-venous ecmo

## Abstract

Acute severe asthma, or status asthmaticus, is a life-threatening condition unresponsive to conventional therapies such as inhaled β-agonists, corticosteroids, and non-invasive ventilation. We report a case of a 41-year-old male with refractory bronchospasm and profound respiratory acidosis despite aggressive medical management. Initial extracorporeal CO₂ removal (ECCO₂R) via a continuous renal replacement therapy (CRRT) system was unsuccessful due to blood flow limitations. The patient was transitioned to venovenous extracorporeal membrane oxygenation (VV ECMO), which led to rapid improvement in gas exchange and stabilization of dynamic hyperinflation. Over the next several days, bronchospasm resolved, ventilator settings were optimized, and the patient was successfully weaned off ECMO and extubated. This case highlights the critical role of extracorporeal therapies, particularly VV ECMO, in managing severe asthma exacerbations refractory to conventional treatment, allowing lung rest and improving outcomes. Emerging evidence supports the efficacy of these modalities, warranting further research to standardize their use in status asthmaticus.

## Introduction

Acute severe asthma, formerly referred to as status asthmaticus, is a critical, life-threatening asthma exacerbation that does not respond to standard treatments, including repeated doses of beta-agonist therapy or subcutaneous epinephrine [1,2]. The refractory bronchospasm can result in hypercarbia, hypoxemia, altered mental status, respiratory failure and eventually death [3]. The pathophysiology involves severe bronchoconstriction, airway inflammation, and hyper-reactivity, leading to significant airflow obstruction, impaired ventilation, and oxygenation [4]. According to the CDC, the asthma death rate in 2021 was 10.6 per million (n=3,517) [5]. Approximately 4% of asthma patients require intubation and mechanical ventilation. The reported in-hospital mortality rate for intubated patients can reach up to 7% [6].

Mechanical ventilation is often required to manage asthmatic patients who deteriorate despite aggressive management; however, it can have deleterious effects due to worsening dynamic hyperinflation and increase intrathoracic pressure [6]. Dynamic hyperinflation is defined as the progressive increase in end-expiratory lung volume above the resting functional residual capacity due to incomplete lung emptying during expiration. This can lead to increased intrathoracic pressure, reduced venous return, and subsequent hemodynamic compromise, including systemic hypotension and barotrauma [7]. When conventional therapies fail, including high-dose beta-2 agonists, magnesium sulfate, systemic corticosteroids, and heliox, advanced extracorporeal support techniques such as extracorporeal carbon dioxide removal (ECCO₂R) and extracorporeal membrane oxygenation (ECMO) may be considered [8,9]. 

ECCO₂R, which operates at lower blood flow rates, primarily aims to remove carbon dioxide and is particularly useful in managing refractory hypercapnia without significantly affecting oxygenation. ECMO, on the other hand, provides comprehensive cardiopulmonary support by both oxygenating the blood and removing carbon dioxide, thus allowing the lungs to rest and recover [8,10]. Venovenous ECMO (VV ECMO) is typically preferred for respiratory support, as it effectively manages severe hypoxemia and hypercapnia [10,11]. The Extracorporeal Life Support Organization (ELSO) registry data indicates that asthma is associated with excellent outcomes for patients requiring ECMO support, with survival rates to hospital discharge as high as 83.5% [9].

The case of a 41-year-old male with severe asthma exacerbation illustrates the complexity of managing refractory bronchospasm and profound respiratory acidosis, necessitating innovative interventions such as ECCO₂R and VV ECMO. This report details the clinical course, diagnostic challenges, and multidisciplinary management of a patient presenting with acute severe asthma exacerbation complicated by dynamic hyperinflation, ventilator desynchrony, and progressive hypercapnia. We implemented advanced modalities, including VV ECMO and Heliox, alongside conventional pharmacologic and ventilatory strategies, to stabilize the patient. Additionally, we employed targeted interventions for refractory bronchospasm and optimized strategies to improve patient outcomes in this severe, life-threatening asthma exacerbation.

## Case presentation

A 41-year-old male with a significant past medical history of tobacco use disorder and childhood asthma (with no baseline pulmonary function tests available in our health system) presented with progressively worsening dyspnea and wheezing over several days. The initial presentation was preceded by upper respiratory tract symptoms, including cough, rhinorrhea, sore throat, myalgia, and arthralgia, which evolved into shortness of breath, fever, and productive cough with yellow sputum. The patient reported increased use of his prescribed asthma inhaler without symptomatic relief and denied recent exposure to sick contacts.

**Figure 1:**
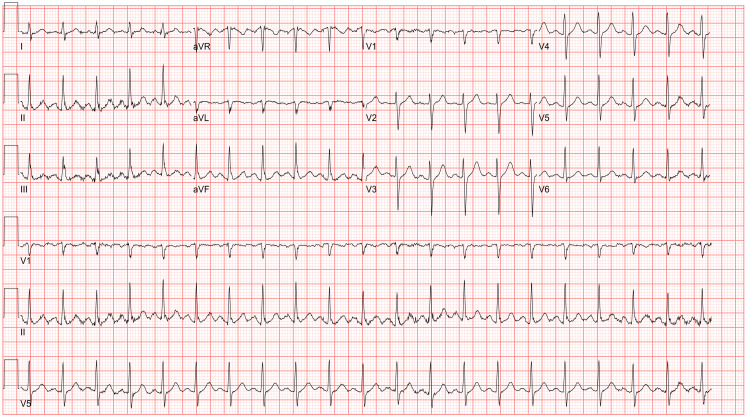
Electrocardiogram Showing Sinus Tachycardia Without Acute Ischemic Changes The electrocardiogram demonstrates sinus tachycardia with a ventricular rate of 123 beats per minute, PR interval of 146 milliseconds, QRS duration of 78 milliseconds, and QTc of 440 milliseconds.

On initial evaluation at an outside facility, the patient exhibited respiratory distress with pronounced expiratory wheezing. An initial diagnostic workup, including COVID-19, respiratory syncytial virus (RSV), and influenza swabs, returned negative. Electrocardiography (EKG) demonstrated sinus tachycardia without acute ischemic changes (Figure 1). Initial venous blood gas (VBG) analysis was unremarkable, and chest radiography showed no acute cardiopulmonary pathology. The patient was initiated on bilevel positive airway pressure (BiPAP) therapy due to his labored work of breathing and received nebulized bronchodilator treatments, intravenous methylprednisolone, and magnesium sulfate. Despite these interventions, his work of breathing remained markedly labored.

Subsequent VBG findings after two hours included a pH of 7.34, partial pressure of carbon dioxide (PCO₂) of 46 mmHg, and bicarbonate of 29 mmol/L. Subsequent arterial blood gas (ABG) analyses in consecutive two hours revealed worsening respiratory acidosis, with the pH declining from 7.33 to 7.17 and PCO₂ rising from 37 mmHg to 60 mmHg. Due to the failure of non-invasive ventilation, persistent respiratory distress, and respiratory acidosis, the patient underwent rapid sequence intubation and was transferred to a tertiary care center.

The patient was commenced on continuous albuterol nebulization and maintained on empiric intravenous methylprednisolone (60 mg every eight hours). Empiric antibiotic therapy with piperacillin-tazobactam was initiated, although his respiratory viral panel returned positive for rhinovirus. Despite these measures and additional ipratropium treatments, the patient remained in significant bronchospasm with persistent wheezing. Lactate levels escalated from 5 to 7 mmol/L, and profound pulsus paradoxus was observed, with systolic blood pressure decreases exceeding 50 mmHg during inspiration.

**Figure 2:**
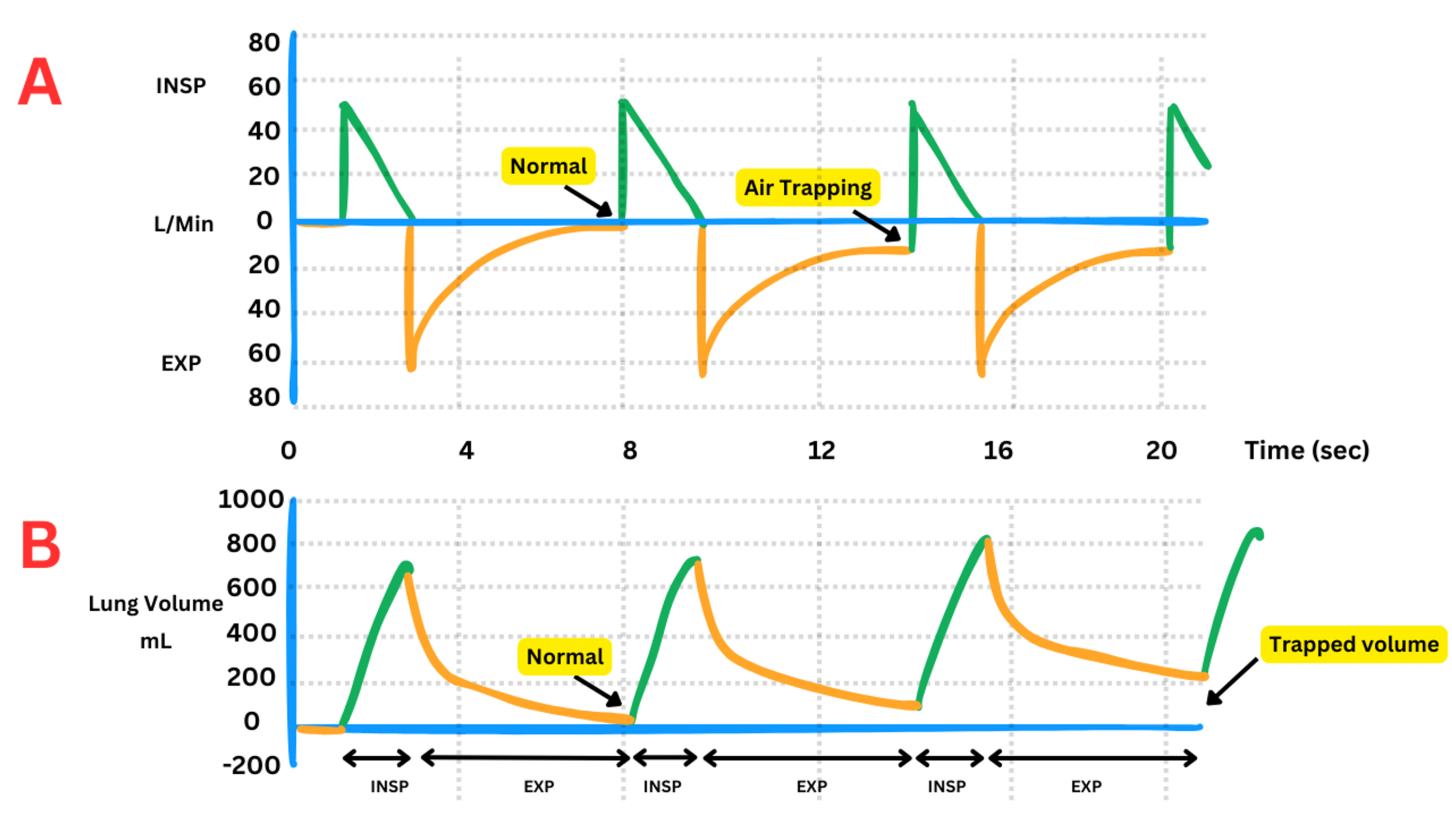
Schematic Representation of Dynamic Hyperinflation in Respiratory Mechanics The figure illustrates dynamic hyperinflation using ventilator waveforms: A: Ventilator flow-time graph displaying inspiratory (INSP) and expiratory (EXP) flow rates. Normal expiratory flow transitions into impaired flow with air trapping, characteristic of dynamic hyperinflation. B: Ventilator volume-time graph showing lung volume changes. The progressively increasing trapped volume is a result of incomplete expiration and airflow limitation.

Upon admission to the tertiary facility, the patient was placed on volume control ventilation, with initial settings of 580 mL tidal volume, positive end-expiratory pressure (PEEP) of 5 cm H₂O, and respiratory rate of 20 breaths/minute. His peak airway pressures were elevated, reaching 40 cm H₂O with a plateau pressure of 24 cm H₂O, indicating significant airway resistance. The patient had significant ventilator dsynchrony with auto-peeping secondary to dynamic hyperinflation (Figure 2). The ventilator setting was changed to pressure control. He, however, continued to take large tidal volumes with continued auto-peeping and worsening respiratory acidosis.

Given the ongoing respiratory decline, intravenous ketamine and midazolam were administered for sedation. Continuous paralytic infusion was initiated. The patient's tidal volumes decreased with improvement in dynamic hyperventilation and hemodynamics. However the patient became profoundly acidotic with a PCO₂ of 120, suggestive of refractory bronchospasm.

At this point, a decision was made to start the patient on ECCO₂R. Given our previous experience of doing ECCO₂R using the continuous renal replacement therapy (CRRT) pump, a dialysis line was placed, and ECCO₂R was initiated via the CRRT machine. The patient was also started on Heliox 80/20. However, severe hypercapnia and respiratory acidosis continued due to extreme bronchospasm even with tidal volumes as low as 200 cc.

The blood flow in the CRRT was increased, but due to high filter pressure, we were unable to go above 350 cc/min. The CRRT filter was the PrismaFlex (Baxter, Deerfield, IL, USA), which has limited flow. Our institution did not stock the PrismaLung (Baxter), which is capable of taking flows up to 600 cc/min needed for optimal CO₂ removal.

**Figure 3:**
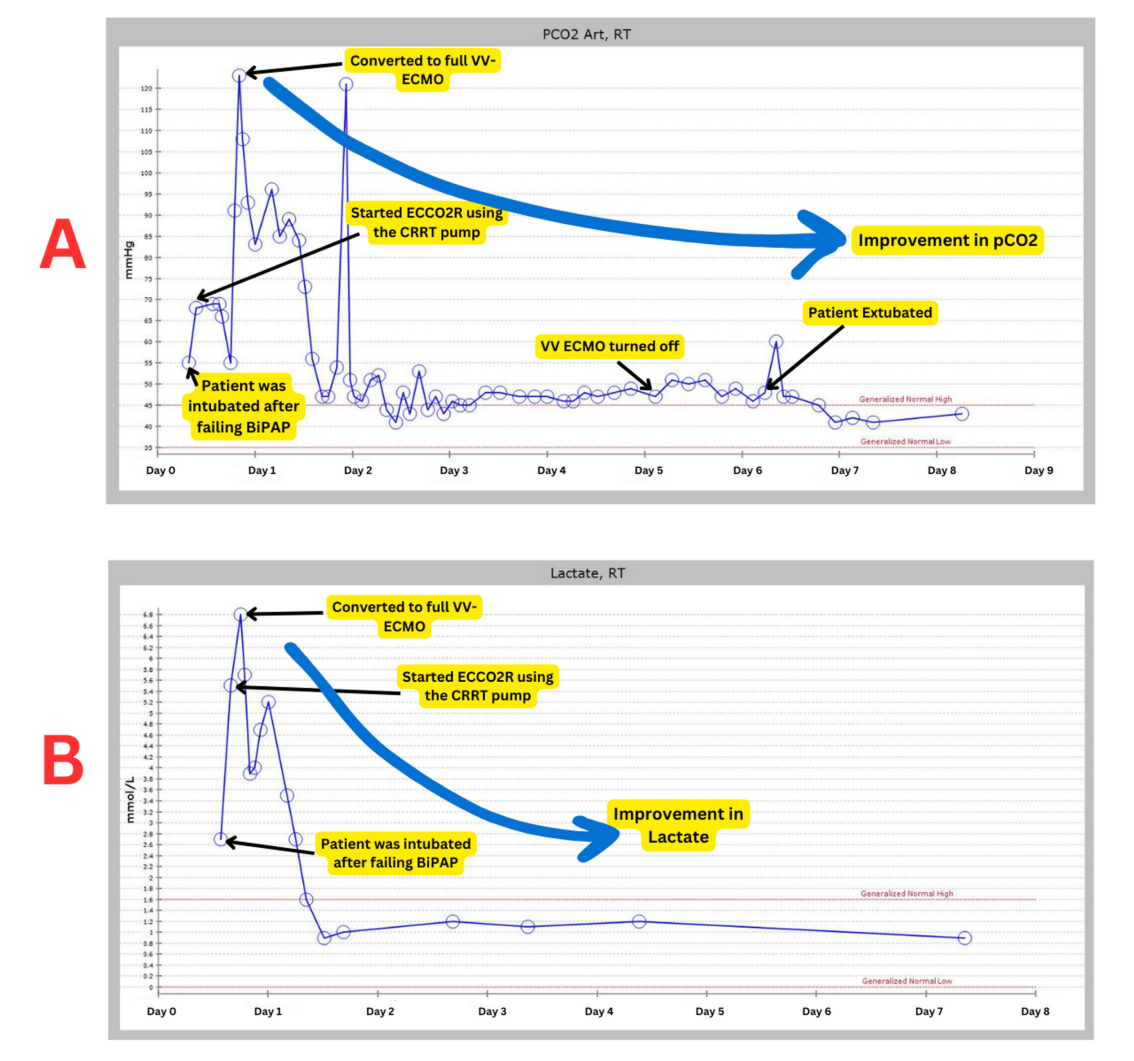
Temporal Trends of Arterial pCO₂ and Lactate Levels During ECMO Therapy in a Patient with Refractory Status Asthmaticus Temporal trends of arterial pCO₂ (A) and lactate (B) during ECMO therapy. Key events include intubation, ECCO₂R initiation, and conversion to full VV ECMO. “RT” indicates blood gas analysis performed by a respiratory therapist. pCO₂: partial pressure of carbon dioxide, ECMO: extracorporeal membrane oxygenation, VV ECMO: venovenous ECMO, ECCO₂R: extracorporeal CO₂ removal, BiPAP: bilevel positive airway pressure, CRRT: continuous renal replacement therapy

**Figure 4:**
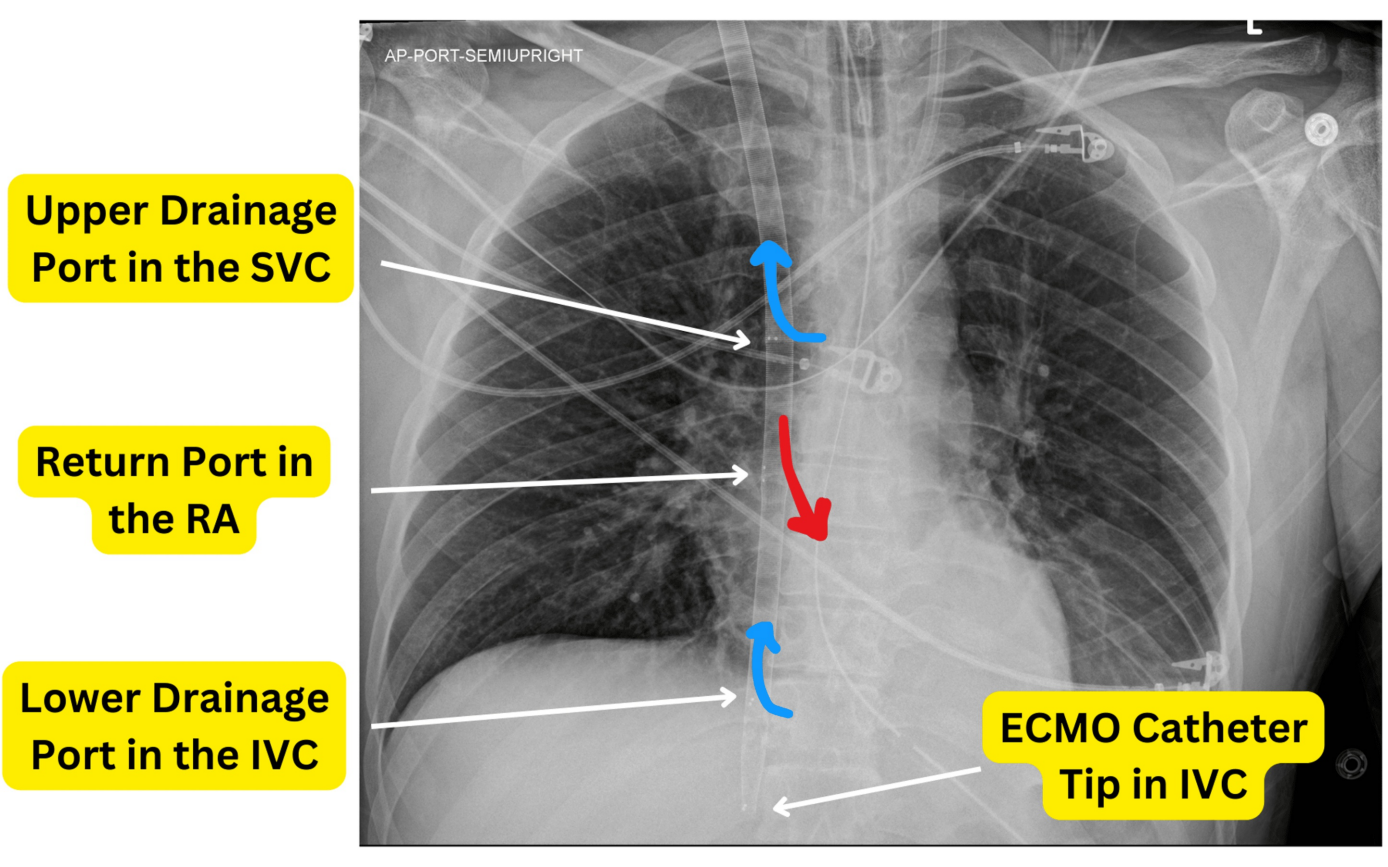
Chest X-Ray Demonstrating Placement of Crescent Dual-Lumen Single-Site VV ECMO Cannula via Right Internal Jugular Vein The chest X-ray illustrates the proper placement of the Crescent Dual-Lumen Single-Site VV ECMO Cannula via the right internal jugular vein. The drainage ports are located in the superior vena cava (SVC) and inferior vena cava (IVC), while the return port is positioned in the right atrium (RA) directed toward the tricuspid valve. ECMO: extracorporeal membrane oxygenation, VV ECMO: venovenous ECMO

Given the worsening clinical status and profound respiratory acidosis, a decision was made to start the patient on full VV ECMO support for optimal CO₂ removal. A 28 FR Crescent ECMO cannula (Medtronic, Minneapolis, MN, USA) was placed in the right internal jugular, and the patient was started on VV ECMO support. Blood of 4 liters was achieved. Initially, we started with a low sweep of 1 liter per minute and slowly, over the next 24 hours, achieved a sweep flow of 5 liters per minute. The CO₂ improved from over 100 to 45, and the pH normalized. The trends in arterial pCO₂ and lactate levels during the course of ECMO therapy are illustrated in Figure 3. The placement of the Crescent Dual-Lumen Single-Site VV ECMO Cannula via the right internal jugular vein is demonstrated in Figure 4, as visualized on chest X-ray.

The ventilator setting was adjusted to have a tidal volume of 200 cc to allow the continuous albuterol to reach the small airways. This required pressure support of 20 cm of H_2_O and produced a peak airway pressure of 45 with a plateau pressure of 25. Over the next several days, the patient's bronchospasm improved, and peak airway pressure declined in the same ventilator setting. The patient could take larger tidal volumes with decreased peak airway pressure, signifying improvement in airway resistance. The patient's clinical parameters before and after ECMO initiation are summarized in Table 1.

**Table 1:** Patient Clinical Status Before and After ECMO Initiation Values in brackets represent the interquartile range (IQR). pCO₂: partial pressure of carbon dioxide, PO₂: partial pressure of oxygen, FiO₂: fraction of inspired oxygen, ECMO: extracorporeal membrane oxygenation, VV ECMO: venovenous ECMO, ECCO2R: extracorporeal CO2 removal

Variable	Pre-ECMO	48 hours after ECMO initiation	48 to 96 hours after ECMO initiation
Ventilator Settings and Measurements	-	-	-
Peak pressure (cm H₂O)	41 (37-45.5)	35 (35-35)	23 (21-24)
Plateau pressure (cm H₂O)	23 (23-24)	23 (21-23.5)	20.5 (19.25-21)
Respiratory rate (breaths/min)	12 (12-15)	11 (10-12)	12 (12-12)
Tidal volume (mL)	489 (438-651.25)	336 (272.25-385.5)	678.5 (595.75-743.25)
Minute volume (L/min)	5.35 (4.68-5.5)	4.35 (2.78-5.02)	8.25 (7.95-9.38)
FiO₂ (%)	75 (65-80)	50 (50-50)	50 (50-50)
Arterial Blood Gas	-	-	-
pH	7.09 (7.08-7.15)	7.42 (7.37-7.44)	7.5 (7.47-7.5)
PCO₂ (mmHg)	90 (84-95)	47 (45-51.25)	47 (47-49)
PO₂ (mmHg)	87 (81-110)	71.5 (64.75-75.25)	79 (75.5-88.5)
Bicarbonate (mmol/L)	28 (26-29)	30.5 (30-31)	37 (34.5-38)
Lactate (mmol/L)	4.35 (3.8-5.32)	1.1 (1-1.2)	1.05 (0.98-1.13)
VV ECMO Parameter	-	-	-
Gas Sweep (L/min)	NA	5 (4-6)	2.5 (1-5)

The patient's ICU course was complicated by Moraxella catarrhalis ventilator-associated pneumonia. He had been placed on prophylactic antibiotics with piperacillin-tazobactam, which was changed to cefepime based on the cultures.

Over the next several days, patients showed significant improvement. The ECMO sweep was slowly turned down, and patient tidal volumes continued to increase even with decreased pressure support on the ventilator. The sweep was turned to 0, and the patient was able to maintain adequate ventilation without ECMO support. On hospital day 8, the ECMO was weaned entirely off and decannulated. Steroid therapy was tapered from 60 mg every eight hours to twice daily. The patient's condition showed significant improvement, evidenced by reduced peak airway pressures, normalized pH (7.4), and CO₂ levels in the mid-40s.

The following day, the patient had a sedation holiday that indicated improved neurological status. He was placed on a weaning trial and successfully extubated. On the next day, he was transferred to the medical floor in stable condition. Post-extubation, the patient experienced hyperactive delirium, which was managed with gradual tapering of benzodiazepines and initiation of olanzapine. On day 13, the patient was discharged home on room air.

## Discussion

Status asthmaticus represents a severe, life-threatening exacerbation of asthma that is unresponsive to standard treatments, including inhaled β-agonists and systemic corticosteroids. Management of this condition often necessitates escalation to adjunct therapies such as intravenous β-agonists, magnesium sulfate, and noninvasive ventilation [12]. In cases where these measures fail, more advanced interventions, including mechanical ventilation and extracorporeal treatments, may be required. ECMO has emerged as a critical rescue therapy for patients with refractory status asthmaticus, providing both respiratory and hemodynamic support while minimizing ventilator-associated lung injury [8,10]. Additionally, ECCO₂R systems have shown promise in managing refractory hypercapnic respiratory failure associated with status asthmaticus, offering an alternative to traditional ECMO with potentially fewer complications [13,14]. These extracorporeal treatments, while not definitive therapies, serve as vital bridges to recovery by stabilizing gas exchange and allowing time for the resolution of the underlying asthma exacerbation.

ECCO₂R plays a significant role in the management of severe asthma, particularly in cases of refractory status asthmaticus where conventional treatments fail to control hypercapnia and respiratory acidosis. ECCO₂R provides a means to remove excess CO₂, thereby correcting hypercapnia and acidosis, and allowing for reduced ventilatory pressures and volumes, which can minimize ventilator-induced lung injury. ECCO₂R systems typically operate at low blood flow rates ranging from 200 to 500 mL/min, which is sufficient to achieve significant CO₂ removal while minimizing the risks associated with higher flow rates [13-16]. The types of filters used in ECCO₂R systems are crucial for effective CO₂ removal. These systems often employ large surface area membrane lungs, such as those with a surface area of 1.8 m², which enhance gas exchange efficiency. For instance, the Hemolung Respiratory Assist System (LivaNova, Houston, TX, USA), a low-flow ECCO₂R device, has been shown to effectively manage refractory hypercapnic respiratory failure in status asthmaticus, achieving CO₂ removal rates of approximately 82.5 ± 15.6 mL/min [13,15-17]. A retrospective review by Bromberger et al. demonstrated that venovenous ECCO₂R significantly improved blood gas values, reduced peak airway pressures, and decreased the need for vasopressors within 24 hours of initiation in patients with status asthmaticus. The study reported a 100% survival rate to hospital discharge, with 76.9% of patients successfully extubated while on ECCO₂R support [14]. In our case, we were unable to successfully remove CO₂ using the PrismaFlex CRRT machine. The primary limitation was the blood flow rate, which was restricted to 350 mL/min. This limitation arose due to the absence of the PrismaLung filter in our institution's stock at that time, which is specifically designed for this indication and can achieve flow rates of up to 600 mL/min. Due to the same reason, the patient was eventually converted to VV ECMO for CO₂ removal.

There are no standard indications for the use of ECMO in severe asthma patients, but the presence of persistently high ventilator peak pressures, refractory respiratory acidosis, or hemodynamic instability have been suggested as justification to initiate ECMO [11]. A retrospective observational cohort study by Zakrajsek et al. that included 13,714 patients showed that ECMO was associated with reduced mortality in the covariate-adjusted (OR, 0.33; 95% CI, 0.17-0.64; P = .001) compared to the non-ECMO group but not with decreased ICU length of stay (LOS), hospital LOS, or time receiving invasive ventilation [11]. Yeo HJ et al. used the Extracorporeal Life Support (ECLS) Organization Registry to measure survival to hospital discharge, complications, and clinical factors associated with in-hospital mortality for asthmatics treated with ECMO. They included 272 people treated with ECMO for asthma between 1992 and 2016. They found that the weaning success rate was 86.7%, and the rate of survival to hospital discharge was 83.5%. They also found that ventilator settings, including rate, fraction of inspired oxygen (FiO₂), peak inspiratory pressure (PIP), mean airway pressure, and driving pressure significantly improved after ECMO initiation [9]. Similarly, Mikkelsen et al. used the ECLS registry to compare ECLS mortality in asthmatics versus non-asthmatic groups. The study population included 1,257. They found that a total of 83.3% of asthmatics survived to hospital discharge compared with 50.8% of non-asthmatics (n=1,233) [odds ratio (OR) favouring survival for asthmatics=4.86, 95% confidence interval (CI) 1.65-14.31, p=0.004]. This remained valid even when controlling for potential confounders [18].

ECCO₂R, while increasingly used, is not yet FDA-approved for status asthmaticus. It has been utilized under Emergency Use Authorization (EUA) during the COVID-19 pandemic for specific cases of refractory hypercapnic respiratory failure [19]. The lack of high-quality randomized controlled trials and the frequency of complications have limited its broader approval [20,21]. Also, the FDA has not granted specific approval for the use of VV ECMO in status asthmaticus; the use of VV ECMO in this context is based on clinical judgment and the extrapolation of its benefits in other forms of severe respiratory failure [22].

## Conclusions

This case underscores the importance of VV ECMO as a life-saving intervention in refractory status asthmaticus, enabling effective CO₂ clearance, stabilization of respiratory acidosis, and lung rest. While ECCO₂R offers promise, equipment limitations may necessitate escalation to ECMO in severe cases. However, ECMO involves risks, including bleeding, thrombosis, and infection, and should be used only after careful multidisciplinary review at experienced centers.
